# Assessment of Adverse Childhood Experiences in the South Bronx on the Risk of Developing Chronic Disease as Adults

**DOI:** 10.7759/cureus.43078

**Published:** 2023-08-07

**Authors:** Alexander Njoroge, Masood A Shariff, Hira W Khan, Victor Gordillo, Brian Eclarinal, Jose Vargas, Mohammad Faiz, Moiz Kasubhai, Tranice Jackson

**Affiliations:** 1 Department of Internal Medicine, NYC HHC (New York City Health and Hospitals Corporation) Lincoln, Bronx, USA

**Keywords:** odds ratio, social determinants of health (sdoh), high risk behavior, chronic disease, adverse childhood experience

## Abstract

Background

Adverse childhood experiences (ACEs) have a negative impact on health outcomes. Using a cross-sectional study design, our objective was to identify the prevalence of ACEs among residents of South Bronx and the increased relationship between such childhood stressors and the prevalence of both chronic disease and modifiable high-risk behavior in adulthood.

Methods

We recruited patients from a hospital-based, adult primary care clinic in the metropolitan area of the South Bronx. A prospectively designed, observational study recruited patients in a consecutive fashion to conduct a cross-sectional survey between September 2017 and January 2018. The demographic representation comprises a low socioeconomic sector of urban New York City, with low education and immigrant population. A modified ACE questionnaire that included nine ACE categories (Physical Abuse, Sexual Abuse, Household Substance Abuse, Separation from Parents, Incarcerated Household Member, Parental Separation/Divorce, and Bullying) in addition to questions on demographics, high-risk behavior, and diagnosis of chronic disease. Our primary objective was to gather the incidence of ACEs organized by domains. Secondary objectives were to demonstrate any expected increase (as odds ratios (ORs)) in chronic disease or maladaptive social habits when compared to patients with no ACEs within the cohort. The OR for the associations was calculated with logistic regression. Individual logistic regression models for each chronic disease, high-risk behavior, and demographics were used to measure the exposure response of the nine ACE categories.

Results

A total of 454 patients completed the survey. The average age was 53.1±14.2 years, and females were 49% of the sample. Hispanics were at 61% followed by Blacks at 34%. Participants reported high-risk behavior at 24%, had a high prevalence of chronic illness (82%), and had ACE events at 70%. We found a significant relationship between ACE events and having a chronic disease diagnosis and engagement in high-risk behavior with higher odds of reporting chronic illnesses among participants with exposure to childhood stressors (OR 1.26, 95% confidence interval 1.1-1.5, p=0.002). Of the nine ACE categories, many were independently associated with one or more chronic diseases in adulthood.

Conclusion

According to our survey data, ACE events in our patient population were more prevalent (30% with four or more exposures), higher than the proposed average of one out of six Americans with four or more exposures nationally according to national statistics. These childhood stressors appeared to have a strong association with the development of high-risk behavior and chronic illnesses.

## Introduction

Adverse childhood experiences (ACEs) have been shown to contribute significantly to negative adult health behaviors and health outcomes. In prospective cohort studies, people exposed to multiple childhood traumatic stressors were at increased risk of premature death compared to those without ACEs [1]. Another investigation has linked ACE to early death and yet others have linked ACE with depressive disorders [2], diagnoses of cancer [3], chronic obstructive pulmonary disease (COPD) [4], premature mortality [5], self-reported sleep disturbances [6], cardiovascular health [7], and high-risk behaviors [8]. Studies have also investigated the association between adverse childhood events and neurological damage, with significant effects on social behavior, affect, and memory [9]. Additionally, strong relationships have been demonstrated between ACEs and risky sexual behaviors, substance use, and aggression [8,10].

Ongoing research continues to highlight the burden of ACEs and their impact on adults, specifically between the associations of ACE and the impact they play in the high-risk behavior and outcomes of these patients with chronic disease. In the United States, an estimated 65% to 87% of adults have experienced at least one ACE in their lifetime [3].

ACE, alongside social determinants of health (SDOH) and environmental factors, all have been proven to influence a person's health [11]. SDOH is another facet that brings to light the impact environment plays on an individual’s well-being, quality of life, and health. SDOH includes gender, social and economic opportunity, food security, social interactions and relationships, and quality of education [12]. Deficits in these elements with exposure to ACE have an impact on healthy development in childhood. In adulthood, this recognition of the deficits highlights the intervention a primary care provider could play in generating a link with the community liaisons in addressing the needs awareness of the resources. As adults that present with multiple comorbidities, primary care settings could elucidate ACE impact and address modifiable risk factors through shared decision-making, in addition to added SDOH inquiries to connect patients with available services in an attempt to improve current environmental deficits, thus engaging in chronic health with a multidimensional care approach.

The South Bronx is the congressional district with the lowest socioeconomic standing per capita in the country, with high rates of substance use and other risk factors for health and chronic diseases [13-16]. To our best knowledge, ACE events have not been measured in this community. This study aims to measure individual specific ACEs in a population of clinic patients along with their existing health conditions/morbidities in order to estimate any associated risk between specific ACE types and subsequent likely negative health diagnoses or substance use.

This article was previously posted to the medRxiv preprint server on May 16, 2023.

## Materials and methods

Methods

The Institutional Review Board of Lincoln Medical Center approved the study (IRB#16-009), and data collection was conducted in a single-center, outpatient adult primary care clinic in the South Bronx, NY. Eligibility criteria included age over 18 years and electronic medical record verification of care being received at the study center for at least one year. The recruitment was a consecutive sampling of patients that presented to the clinic for primary care appointments. They were called a day prior to the visit to introduce the study and gauge their willingness to participate in answering the survey questionnaire prior to or following their clinic appointment. Research staff conducted face-to-face interviews and administered a modified ACE questionnaire (discussed below). Cognizant of the sensitive nature of the interview, all research staff underwent training on interview techniques and followed a protocoled script to discuss with prospective patients, including engagement, confidentiality, the research question, and the goals of the study. The questionnaire was pre-tested among medical students and outpatient clinical staff for clarity and timing. The study and its goals were reviewed again with the patients presented for their primary care appointment, who consented and were interviewed in an enclosed space, with a suitable level of privacy. Upon completion, survey data were entered into an electronic database and de-identified.

More than 50% of the South Bronx patient population are largely of Hispanic descent/Spanish speakers. Our research staff consisted of certified native Spanish speakers who engaged these patients and conducted their surveys.

Questionnaire design

According to the American Academy of Pediatrics (AAP), there are 10 recognized adverse childhood events [17]. These stressors were originally formulated using information from the first ACE study [1] and include Emotional Abuse; Mother Treated Violently; Physical Abuse; Household Substance Use; Sexual Abuse; Household Mental Illnesses; Emotional Neglect; Parental Separation or Divorce; Physical Neglect; and Incarcerated Household Member. Our survey design was developed institutionally, with input from the Psychiatric and Pediatric departments, with emphasis on two ACE definitions/domains: Abuse and Household Dysfunction. We modified wording in the ACE questions about sexual abuse, verbal abuse, and mother treated violently; these changes were introduced as a team to customize the survey to be more culturally sensitive to our population. Adjustments also included the addition of four categories: parental separation or divorce, bullying in school, separation from parents, and living with foster parents, as these were not part of the standard ACE questionnaire. These categories were included following the review of the AAP publications on childhood stressors [18]. The AAP has emphasized all four categories as significant causes of childhood distress, especially in the early years [17]. The complete ACE study tool is shown in the Appendices.

**Figure 1:**
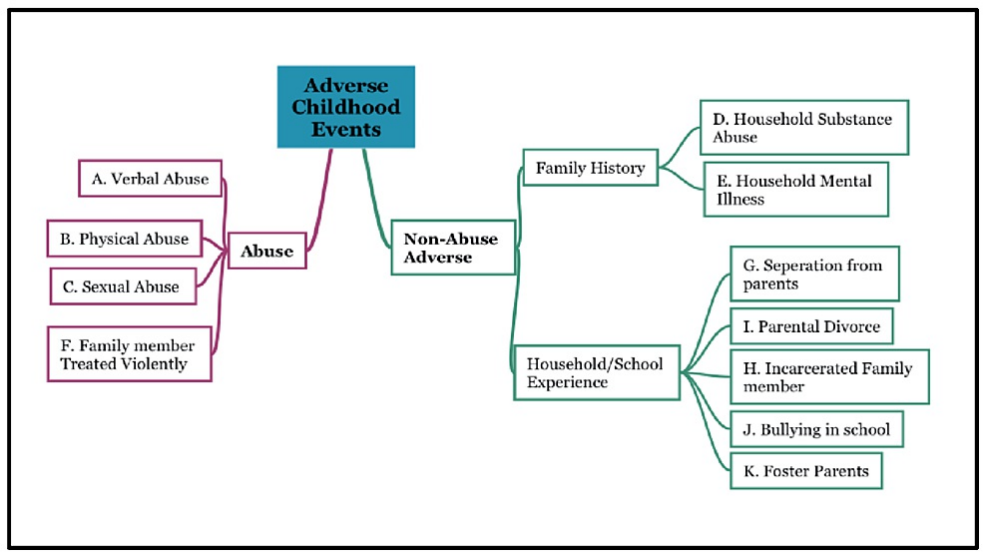
Distribution of ACE definitions and categories ACE: adverse childhood experience

The study questionnaire was modified from the original ACE study [1], the Adverse Childhood Experience Questionnaire for Adults, and other formats of the survey [19] were utilized referring to the subject’s life before 18 years of age. There were three categories within the Abuse definition: A - Verbal abuse, B - Physical abuse, and C - Sexual abuse. Eight categories within the Household dysfunction definition: D - Household substance use, E - Household mental illness, F - Family member treated violently, G - Separation from parents, H - Incarcerated family member, I - Parental separation or divorce, J - Bullying in school, and K - Foster parents. Figure 1 illustrates the distribution of ACE categories.

Studies have been published using various combinations of the original questionnaire. There are four questions within our abuse definition that inquired about verbal abuse (Category A), physical abuse (Category B), and sexual abuse (Category C). Category B (physical abuse) is subdivided into two questions. Under household dysfunction, family members were treated violently, household substance use, household mental illness, parental separation or divorce, and incarcerated household members were inquired. Category D (Household Substance Use) is subdivided into two questions. Category F (Family member treated violently) is subdivided into four questions, for a total of 13 household dysfunction questions. All responses were binary, and participants were marked as exposed if they responded “yes” to any of the subdivided category questions. The measure of exposure, or ACE score, is the sum of positive categories; and the scores ranged from 0 (unexposed) to 11 (exposed to all categories).

Risky behavior and chronic disease assessment

We added questions to the prior survey about participants’ subjective health (i.e., how would you describe your health?) and medical diagnoses (ie. has a doctor, nurse, or another healthcare provider ever told you that you have asthma?). Chronic illnesses were assessed by collecting a history of hypertension, dysglycemia/diabetes, hyperlipidemia, heart attack event, angina/coronary heart disease (CHD), stroke, asthma, COPD, congestive heart failure (CHF), hepatitis, and poor mental health/antipsychotic use (in the past 30 days).

We also included three questions assessing high-risk behavior such as smoking status, alcohol, and substance or drug use. High-risk behavior was screened with three pertinent questions on smoking history (daily, somedays, quit, or none), daily alcohol use (one or more drinks as per the CDC definition [20]), and substance use (marijuana, heroin, cocaine, methamphetamine, or prescription drugs).

Demographics assessed

Demographic information was assessed through the use of 11 questions on age, gender, race, primary language, place of birth, years in the United States (US), insurance type, education, marital status, and employment status/description. The questionnaire is attached in the Appendices.

Statistical analysis

Our sample size was 454, and we used a confidence interval of 95% and an alpha error of 0.05 to evaluate associations between childhood stressors and health outcomes & childhood stressors and risky behavior. We used multiple logistic regression to assess significant relationships between various chronic illnesses, total ACE score, specific ACE categories, and other covariates. The odds ratio (OR) for the associations was calculated by logistic regression using SPSS Statistics for Windows Version 15 (SPSS Inc, Chicago, Illinois). The 95% confidence interval (CI) and P-value for each OR were tabulated. P<.05 was deemed significant. Individual logistic regression models for each of the 11 health variables listed were used to measure the exposure-response relationship of ACE scores with health outcomes.

## Results

Demographics

Overall, the number of patients screened on a consecutive basis was 610 in the ambulatory clinic setting and 454 patients consented. The basic demographic breakdown is demonstrated in Table 1. The average age of the participants was 53.1±14.2 years, with a female prevalence of 49%. A majority of the participants were Hispanic (61%), followed by Black (34%), then Others (5%). Other demographics of note for our patient population were Asians and some Whites. The most common primary language was Spanish (56%), and a majority of the foreign-born (76%) had lived in the US for more than 10 years (86%). The insurance under “Other” represented some combination of private/hospital coverage with Medicare and/or Medicaid (64%). Furthermore, 12% had Medicaid alone, and 13% reported no coverage at all. About 51% of the participants stated they had not finished high school (US equivalent). Marital status was distributed with “Never Married” (36%) and “Married” (35%) being the most common responses followed by “Divorced” (12%) and “Widow/Widower” (9%). Overall, 44% of participants were employed and described jobs in varied sectors, and of those working, nearly half reported more than one job (49%). Participants categorized their job type as “semi-skilled” (16%), “service” (13%), and “skilled” (12%).

**Table 1:** Participant characteristics Data is presented as n (frequency, %). *Other insurance includes Private only, Medicare and Private, and Hospital Issued. ACE, adverse childhood experience; GED, general educational development tests.

	Overall	ACE	No ACE	p-value
N	454	317	137	
Age, years (Mean±SD)	53.1±14.2	52.8±14.6	53.7±14.8	0.585
Age (years)				0.548
19-29	33 (7.3%)	23 (7.3%)	10 (7.3%)	
30-39	48 (10.6%)	34 (10.7%)	14 (10.2%)	
40-49	75 (16.5%)	50 (15.8%)	25 (18.2%)	
50-59	116 (25.6%)	88 (27.8%)	28 (20.4%)	
60 and over	182 (40.1%)	122 (38.5%)	60 (43.8%)	
	454	317	137	
Gender				0.994
Male	166 (36.6%)	116 (36.6%)	50 (36.5%)	
Female	223 (49.1%)	156 (49.2%)	67 (48.9%)	
Non-binary	65 (14.3%)	45 (14.2%)	20 (14.6%)	
Race				<0.010
Hispanic	246 (54.2%)	174 (54.9%)	72 (52.6%)	
Black	89 (19.6%)	71 (22.4%)	18 (13.1%)	
Other	119 (26.2%)	72 (22.7%)	47 (34.3%)	
Primary Language				0.001
English	176 (38.8%)	133 (42.0%)	43 (31.4%)	
Spanish	252 (55.5%)	174 (54.9%)	78 (56.9%)	
Other	26 (5.7%)	10 (3.2%)	16 (11.7%)	
Place of Birth				<0.010
US	111 (24.4%)	97 (30.6%)	14 (10.2%)	
Foreign born	343 (75.6%)	220 (64.2%)	123 (35.8%)	
Residence in the US				0.272
>10 years	390 (85.9%)	279 (88.0%)	111 (81.0%)	
5-10 years	22 (4.8%)	13 (4.1%)	9 (6.6%)	
1-5 years	34 (7.5%)	20 (6.3%)	14 (10.2%)	
Less than 1 year	8 (1.8%)	5 (1.6%)	3 (2.2%)	
Insurance Type				0.024
Medicare & Medicaid	34 (7.5%)	16 (5.0%)	18 (13.1%)	
Medicare	19 (4.2%)	13 (4.1%)	6 (4.4%)	
Medicaid	55 (12.1%)	38 (12.0%)	17 (12.4%)	
Other*	289 (63.7%)	212 (66.9%)	77 (56.2%)	
Self-pay	57 (12.6%)	38 (12.0%)	19 (13.9%)	
Education Attained				0.355
Did not finish high school	230 (50.7%)	169 (53.3%)	61 (44.5%)	
Finished high school/GED	84 (18.5%)	58 (18.3%)	26 (19.0%)	
Some college	89 (19.6%)	56 (17.7%)	33 (24.1%)	
College graduate	49 (10.8%)	32 (10.1%)	17 (12.4%)	
Never attended	1 (0.2%)	1 (0.3%)	0	
Marital Status				0.025
Never married	165 (36.3%)	121 (38.2%)	44 (32.1%)	
Married	160 (35.2%)	97 (30.6%)	63 (46.0%)	
Divorced	55 (12.1%)	42 (13.2%)	13 (9.5%)	
Partnership	12 (2.6%)	11 (3.5%)	1 (0.7%)	
Widow/Widower	39 (8.6%)	29 (9.1%)	10 (7.3%)	
Separated	22 (4.8%)	17 (5.4%)	5 (3.6%)	
Refused	1 (0.2%)	0	1 (0.7%)	
Current Employment Status				0.414
Employed for wages	202 (44.5%)	139 (43.8%)	63 (46.0%)	
Retired	81 (17.8%)	54 (17.0%)	27 (19.7%)	
Disabled	58 (12.8%)	49 (15.5%)	9 (6.6%)	
Out of work for more than a year	38 (8.4%)	25 (7.9%)	13 (9.5%)	
Out of work for less than a year	36 (7.9%)	25 (7.9%)	11 (8.0%)	
Homemaker	23 (5.1%)	17 (5.4%)	6 (4.4%)	
Self-employed	14 (3.1%)	7 (2.2%)	7 (5.1%)	
Student	2 (0.4%)	1 (0.3%)	1 (0.7%)	
Job Description (Current/Previous)				0.197
Semi-skilled worker	73 (16.1%)	51 (16.1%)	22 (16.1%)	
Service industry	61 (13.4%)	41 (12.9%)	20 (14.6%)	
Skilled worker	56 (12.3%)	40 (12.6%)	16 (11.7%)	
Sales	23 (5.1%)	15 (4.7%)	8 (5.8%)	
Clerical	10 (2.2%)	5 (1.6%)	5 (3.6%)	
Managerial	5 (1.1%)	4 (1.3%)	1 (0.7%)	
Professional	4 (0.9%)	1 (0.3%)	3 (2.2%)	
Home health aide	1 (0.2%)	0	1 (0.7%)	
Other, variety of jobs	221 (48.7%)	160 (50.5%)	61 (44.5%)	

Social habits

Table 2 presents the participant’s social history, overall health perception, high-risk behavior, and chronic disease status. A majority of the patients stated they never smoked (76%), 9% had quit smoking, and 16% were current smokers. Daily alcohol consumption was reported in 9% of patients, whereas daily substance use/drug use was at 3%. General health self-assessment was “Fair” at 47% and “Good” at 31%. Chronic diseases reported from highest to lowest were: hypertension (59%), hyperlipidemia (48%), dysglycemia/diabetes (43%), asthma (22%), angina/CHD (10%), followed by heart attack, stroke, COPD, hepatitis and CHF. Poor mental health and/or antipsychotic use was 11% for the overall group.

**Table 2:** Participants' current overall general health, high-risk behavior, and chronic disease status Data are presented as n (frequency, %). * Smoking variables were combined to Never smoked, Quit, and Smoker. ACE, adverse childhood experience; CHD, coronary artery disease; CHF, congestive heart failure; COPD, chronic obstructive pulmonary disease.

	Overall	ACE	No ACE	p-value
N/n	454	314	140	
In General, Your Health Is:				0.394
Excellent	39 (8.6%)	27 (8.6%)	12 (8.6%)	
Very Good	14 (3.1%)	10 (3.2%)	4 (2.9%)	
Good	139 (30.6%)	87 (27.7%)	52 (37.1%)	
Fair	215 (47.4%)	153 (48.7%)	62 (44.3%)	
Poor	26 (5.7%)	19 (6.1%)	7 (5.0%)	
Bad	12 (2.6%)	11 (3.5%)	1 (0.7%)	
Not Sure	6 (1.3%)	4 (1.3%)	2 (1.4%)	
Don’t Know	3 (0.7%)	3 (1.0%)	0	
Chronic Disease				
Hypertension	266 (58.6%)	182 (58.0%)	84 (60.0%)	0.766
Hyperlipidemia	219 (48.2%)	157 (50.0%)	62 (44.3%)	0.400
Diabetes	195 (43.0%)	141 (44.9%)	54 (38.6%)	0.317
Asthma	101 (22.2%)	73 (23.2%)	28 (20.0%)	0.271
Angina/CHD	46 (10.1%)	33 (10.5%)	13 (9.3%)	0.968
Heart Attack Event	32 (7.0%)	19 (6.1%)	13 (9.3%)	0.182
Stroke	27 (5.9%)	20 (6.4%)	7 (5.0%)	0.620
COPD	22 (4.8%)	19 (6.1%)	3 (2.1%)	0.083
Hepatitis	23 (5.1%)	14 (4.5%)	9 (6.4%)	0.621
CHF	15 (3.3%)	9 (2.9%)	6 (4.3%)	0.399
High-Risk Behavior				
Alcohol Use	41 (9.0%)	34 (10.8%)	7 (5.0%)	0.082
Mental Health	49 (10.8%)	45 (14.3%)	4 (2.9%)	0.001
Substance Use	15 (3.3%)	14 (4.5%)	1 (0.7%)	0.016
Smoking Status				0.024*
Never Smoked	343 (75.6%)	226 (72.0%)	117 (83.6%)	
Previous Smoker	40 (8.8%)	31 (9.9%)	9 (6.4%)	
Current Smoker	71 (15.6%)	57 (18.0%)	14 (10.2%)	
Smoker - Some Days	33 (7.3%)	21 (6.7%)	12 (8.6%)	
Smoker - Every Day	38 (8.4%)	36 (11.5%)	2 (1.4%)	

ACE data

**Figure 2:**
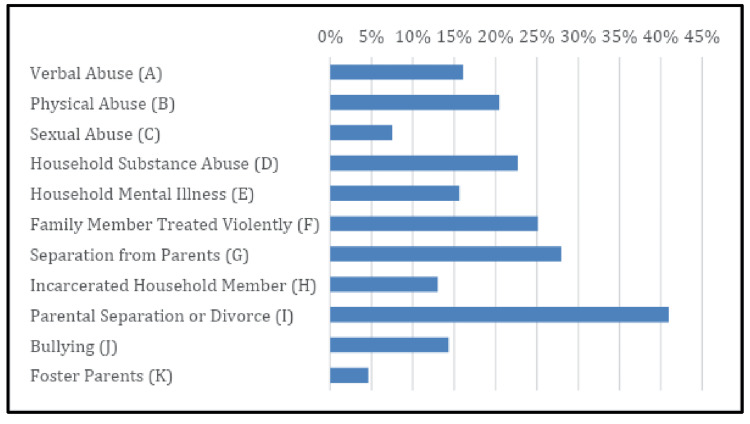
ACE incidence by sub-category ACE, adverse childhood experience.

Figure 2 presents all the ACE components surveyed in the study. The commonest ACEs experienced were as follows: 59% of participants said they experienced “parent separation/divorce” (Category I) as children; followed by “separated from parent” (Category G) (40%) and “family members treated violently” (Category F) (19%). Participants who did not report any ACE events were 43% (n=137) while the remainder (57%; n=317) had a median event of one ACE, and it ranged from 1 to 11 with one event being the majority at 21% overall. The ACE events were summed as total events per participant and the average was 2.1±2.4 events overall.

Subgroup comparison between the “No ACE” versus “One or More ACE” (Table 1) revealed there was no difference in relative risk between men and women. Distribution by race similarly demonstrated no difference between subgroups. Participants born in the US had a higher incidence of ACE versus those born outside the US (87% vs. 64%). More than half (53%) of those who did not finish high school reported one or more ACEs. Those married faced one or more ACEs (31%) while “never married” (38% one or more ACE vs. 32% no ACE) and “divorced” (13% vs. 9%) were higher in the events subgroup compared to no ACE, respectively. Employment status and types of employment were comparable between the groups without significance.

In subgroup comparison (Table 2), the overall health assessment indicated a difference of 10% for self-reported “Good” health between the ACE (28%) and no ACE groups (36%). Smoking every day was also significantly higher in the events group at 12% versus 1% in the no events group (p=0.024). Participants with an ACE event had a higher incidence of both reported mental health (14% vs. 3%, p<0.01) conditions than those with no exposure and substance use (5% vs. 1%, p=0.016).

Odds ratios: by ACE-specific association with chronic disease

The findings demonstrated a high prevalence of both chronic illnesses (80%) and at least one ACE (70%) at baseline within the surveyed population. The incidence of the most common chronic diseases reported was hypertension (59%), hyperlipidemia (48%), and diabetes (43%). Between no ACE and at least one ACE, the burden of chronic disease was the same. Certain ACE categories were correlated with having a larger number of chronic diseases (hypertension/hyperlipidemia/diabetes); they include Sexual Abuse (OR 21.53, 4.14, and 4.65, respectively); Household Substance Use (OR 2.82, 1.80, and 2.05, respectively); Mental Illness (OR 2.95, 2.29, and 2.00, respectively); and Parent’s Separated or Divorce (OR 3.83, 2.53, and 2.18, respectively) (p<0.01). Additionally, Physical Abuse (B) predicted having two chronic diseases (OR 2.52 CI 1.3-5.0, p<0.01). Finally, participants with one or more chronic diseases significantly correlated with Separation from Parents (G) (OR 2.94, CI 1.66-5.22, p<0.01), Incarcerated Household Member (H) (OR 2.61, CI 1.12-6.08, p<0.05), and Bullying (J) (OR 4.84, CI 1.89-12.38, p<0.01). Table 3 lists the odds ratios between chronic disease and ACE categories.

**Table 3:** Odds ratio for the number of chronic diseases and ACE categories Significant *p<0.05 and **p<.01. ACE, adverse childhood experience.

ACE Categories	With One Chronic Disease			With Two Chronic Diseases			With Three Chronic Diseases		
	OR	95% C.I.		OR	95% C.I.		OR	95% C.I.	
A - Verbal Abuse	0.695	0.28	1.73	0.59	0.28	1.27	0.55	0.251	1.22
B - Physical Abuse	2.03	0.894	4.62	2.52**	1.27	5	2.11*	1.06	4.21
C - Sexual Abuse	21.53**	2.62	176.89	4.14**	1.47	11.68	4.65**	1.87	11.54
D - Household Substance Use	2.82**	1.46	5.44	1.80*	1.05	3.08	2.05**	1.19	3.51
E - Household Mental Illness	2.95*	1.26	6.9	2.29*	1.21	4.35	2.01*	1.09	3.7
F - Treated Violently by Family Member	1.43	0.71	2.87	0.86	0.48	1.53	1	0.55	1.8
G - Separation from Parents	2.94**	1.66	5.22	1.80*	1.1	2.96	1.08	0.63	1.86
H - Incarcerated Household Member	2.61*	1.12	6.08	1.68	0.87	3.26	1.2	0.61	2.34
I - Parental Separation/Divorce	3.83**	2.31	6.37	2.53**	1.6	4	2.18**	1.33	3.59
J - Bullying	4.84**	1.89	12.38	1.7	0.86	3.33	2.00*	1.04	3.88
K - Foster Parent	0.737	0.15	3.61	0.7	0.23	2.16	0.23*	0.07	0.87
Constant	0			0.001			0.005		

The odds of having any one of the 11 chronic diseases were higher in respondents with one or more ACEs when compared to individuals with no ACE. The ACE score (number of ACE) also appeared to have a significant relationship with chronic disease (OR 1.26, CI 1.1-1.5, p<0.01); however, there was no association between an ACE score of greater than four and less than four with chronic disease. ACE increases the risk of chronic disease but the risk did not increase with the number of ACE events.

Further analysis involved comparing individual chronic illnesses with ACE scores and ACE categories. Most individual ACE categories showed significant relationships with eight of the 11 chronic illnesses to varying degrees, with the highest odds ratio relating to hypertension, hyperlipidemia, dysglycemia, CHD, stroke, asthma, COPD, and history of mental health/antipsychotic use. High-risk behaviors (alcohol use disorder, substance use disorder, and smoking history) also correlated individually with ACE categories significantly (Table 4).

**Table 4:** Incidence of ACE categories with chronic disease and adverse social habits (number of patients with X condition who had Y category of ACE) Significant *p<0.05 and **p<.010. ACE, adverse childhood experience; CHD, coronary heart disease; CHF, congestive heart failure; COPD, chronic obstructive pulmonary disease.

	ACE Categories										
	Verbal Abuse (A)	Physical Abuse (B)	Sexual Abuse (C)	Household Substance Use (D)	Household Mental Illness (E)	Treated Violently by Family Member (F)	Separation From Parents (G)	Incarcerated Household Member (H)	Parental Separation/Divorce (I)	Bullying (J)	Foster Parent (K)
Hypertension	41	60	29**	59	51*	70	74	36	111	44	11
Hyperlipidemia	36	54*	24**	52	40	56	61	32	89	32	12
Diabetes	32	45*	21*	47	35	49	53	30	75	33	10
Heart attack event	6	7	3	8	8	11	10	4	14	5	1
Angina/CHD	7	14	7*	13	11	14	12	10	19	13**	5*
Stroke	6	7	5*	8	8*	7	9	2	16*	6	1
Asthma	23*	32**	10	29	28**	31	33	15	45	26**	6
COPD	8**	11**	2	9*	6	8	9	6*	11	7*	1
CHF	1	3	2	3	2	5	1	3	5	3	0
Hepatitis	3	3	2	10*	3	6	5	4	8	3	0
Mental Health (> 14 days for the last 30 days)	15**	20**	7	21**	19**	26**	16	7	28*	17**	3
Alcohol - Daily Use	16**	19**	7*	17**	18**	20**	16	9	19	13**	3
Substance Use History	9**	9**	4*	8*	8**	7	10*	7**	12*	8**	5**
Smoking History	27**	32*	10	38**	24*	34	40*	23**	58**	21	6

## Discussion

This study was conducted in an urban, mostly low-income, low-education, immigrant population to assess ACE. In our sample of 454 patients at an urban primary care clinic, 70% had experienced at least one ACE event, and of those, 80% had chronic disease. Having an ACE event/score of at least 1 predicted a current diagnosis of chronic medical disease. This was likely driven by particular subcategories of ACE that showed an impact on chronic disease, mental health, and substance use. We found that the people giving an affirmative response to Parent Divorce/Separation was a commonly reported category in our sample size, followed by Separated from Parent, Family Member Treated Violently, Household Substance Use, and Physical Abuse having more than 20% response rate by the participants for those categories.

Mental health and chronic disease 

There was a significant correlation between chronic illnesses and mental health diseases in all the ACE categories, which has also been shown in other studies with an ACE score of 4 plus [20]. Among our study participants who reported one or more high-risk behaviors, a majority also reported having at least one ACE event, consistent with the established relationship between childhood stressors and high-risk behaviors in other studies [8,21].

ACE vs no ACE

In comparing ACE and no ACE for high-risk behaviors, we found a significant dominance of behaviors such as smoking, alcohol, and substance use in the ACE group. Further, this showed a significant association with the ACE A through K categories.

Results contrary to the existing literature

Our results demonstrated no gender difference for risk of ACE, differing from the increased risk for females demonstrated in most existing literature [22]. The majority of the respondents who reported one or more ACEs were of Hispanic descent, compared to Black descent and others. Cultural perceptions between different ethnicities may influence the recognition and reporting of childhood stressors. Ye et al. suggested that there are variations in cultural influences and certain parenting practices, such as disciplinary actions, which may or may not be perceived as abuse [23]. Furthermore, the reporting of ACEs by individuals of various ethnic or cultural backgrounds may be influenced by their shame or fear of social embarrassment [23]. In our findings, the relationship between ACEs and age groups showed a high number of ACEs reported by individuals older than 45 years, contrary to other ACE studies and surveys, which linked higher ACE scores to younger adults [24].

The chronic diseases in our study were well-distributed between the "more than one ACE" and "no ACE" groups. Hypertension, hyperlipidemia, diabetes, and asthma were on top of the list, as Hispanics and Blacks are predisposed to diabetes and hypertension [22,25]. Although we did not find any significance in participants who had one or more ACE events compared to the ones who had none for chronic disease, there was a correlation between having one or more ACE and a recent history of mental health or use of psychiatric medications for anxiety or depression, which was statistically significant. The participants who experienced ACE were diagnosed by a primary care provider and prescribed medication for mental health. This correlation has been found in other studies where individuals who experienced ACE events were diagnosed with a mental health condition and sought help [23,26]. Mental health impacts socioeconomic factors, influencing the capacity to maintain employment, aggressive behavior, marital issues, and educational attainment [27].

Findings consistent with prior studies

Consistent with previous findings, ACEs prevalent among the residents of South Bronx are associated with certain poor health outcomes, as they have been shown to do in other communities. Having one or more ACEs is likely found among residents of Hispanic descent and older age groups. Sexual abuse, family history of mental health, and bullying were associated with hypertension, hyperlipidemia, and dysglycemia with a higher odds ratio. No significant associations were demonstrated between ACEs and diabetes, heart attack, stroke, heart failure, and hepatitis. The Bronx community comprises many foreign-born, low-income populations. It follows then that they are likely to present with a burden of ACEs from their country of origin. ACE events are more prevalent in low- and middle-income countries owing to limited resources and less social health care [28]. Thus, the idea of being assimilated into the US culture and its correlation with a chronic disease actually could have an underlying basis in immigrants with the “ACE burden” that they have experienced, thus highlighting the added effort the healthcare community needs to put forth while addressing their medical needs.

Future steps

A higher ACE score is correlated with a higher probability of having a chronic disease. Our findings suggest that the health care system should incorporate an ACE questionnaire into their current SDOH screenings in primary care and develop services for patients to address the mental health impact of ACE. One study found that enabling services (such as coordinating care, improving health education, transportation, and access to food, shelter, and health care benefits) found an association with a percentage increase in health center visits, higher probability of getting routine checkups (i.e. flu shots), and patient satisfaction, concluding with the confirmed impact of systematic delivery in health services by reducing access barriers [29]. ACE and SDOH have been shown to intersect, and as a result, have been determined to influence health conditions and behaviors into adulthood; thus it becomes imperative to address both SDOH and ACEs. In line with several other studies that used a similar ACE questionnaire, our study also found that chronic diseases in adulthood are often linked to ACE exposure. As a result of this correlation, it becomes important for physicians to implement processes to systematically address social needs as part of clinical care, especially conditions that potentially have the greatest influence on a patient's health. These processes could include social services, education, one's physical environment, and nutrition.

In childhood development, ACE is balanced by mitigating factors that act as protective features in development that buffer out the negative effects to a degree [30]. The most protective feature is an environment that is supportive and fostered individual relationships through caregivers. ACE in adults does raise a dilemma about how to counteract a response that occurred and impacted early development. Thus, SDOH integration, mental-health referral, and shared decision-making for lifestyle change could alleviate and address current risk factors and environmental stressors and reinstitute a cycle of positive change.

While there are many ways for healthcare providers to help their patients address their underlying social conditions, providers should also begin by systematically screening patients for health-related social needs. This initial screening and discussion with the patient can ultimately impact their health by determining their needs and current socio-economic situation early on. In addition, by working alongside community organizations, providers can help patients who may have difficulty accessing health care by facilitating their capability to acquire housing, food, transportation, and/or other social services. Finally, providers have more influence when it comes to health policy, and they are in a unique position to alter a state and federal government’s ability to prioritize improving health services and facilities for all its residents within its jurisdiction. Through proactive collaboration with community organizations, health care policy changes and changers, and patient education, it becomes possible to potentially disentangle the intersect that exists between ACE and SDOH.

Limitations

There are limitations encountered in this study. First, our findings only represent the South Bronx residents who receive healthcare at a single center. Therefore, residents of the community who were affiliated with other institutions were not adequately represented. Second, the investigation and subsequent results are centered around a modified study tool. Adaptations made to questions on sexual abuse, verbal abuse, and mother treated violently from the original ACE survey included shortened sentence phrasing and rewording, mainly to accommodate translation into various languages encountered in the South Bronx. Our modifications to the ACE survey, however, were not subject to validation by a scrutinized task force, but the survey did reflect the question scheme as in prior publications referenced. Third, the survey inquired about childhood upbringing; therefore, recollection or recall bias may affect the reporting of ACEs. Further, we did find an impact between ACE and mental illness; however, our study did not resolve specific psychiatric illnesses such as anxiety or depression. A history of “poor mental health or antipsychotic drug use” does not give a precise idea of the mental health issues the participants were facing. Because we conducted the survey in this community with a large Hispanic pool of participants with the majority of individuals being foreign-born, the literature search was scarce in capturing this urban region type with the low-income, low-education, immigrant population. This could act as a selection bias, which we couldn't get away from. Finally, our sample size was adequate based on adequate power to conclude associations with chronic diseases significantly. However, possibly due to sampling bias, the limited responses of other chronic illnesses, such as stroke or heart attack, could have been curtailed by recruiting patients from the morning, evening, and weekend clinic sessions. ACE has not been evaluated in the South Bronx region to our knowledge, thus we hope to contribute this information with our research to the overall scientific community and anchor our findings with continued interrogation of ACE associations with other comorbidities such as mental health.

## Conclusions

Our study builds upon the existing body of evidence by investigating the local prevalence and impact of ACEs in the setting of a public hospital that is the primary source of access to health care for many members of the community. Our study found that chronic diseases in adulthood are associated with prior ACE exposure, most notably with hyperlipidemia, hypertension, diabetes, mental illness, or substance use. Such data elucidates the need for physicians to prioritize and incorporate social services, education, physical environment, and nutritional access in overall patient well-being. While there are many ways for healthcare providers to help their patients address their underlying social conditions, we believe a strong and feasible start would be for providers to systematically screen patients for health-related social needs. Accordingly, proactive collaboration with specific community organizations could spur local and larger attempts to eventually rectify the burden of ACE on SDOH.

## Appendices

**Table 5:** Modified ACE questionnaire ACE, adverse childhood experience.

ACE QUESTIONNAIRE				
Verbal Abuse	Category A	Did a parent often swear at you or insult you?	Yes	No
Physical Abuse	Category B	Did a parent or adult often push, grab or slap you?	Yes	No
		Did a parent or adult hit you so hard that you were bruised?	Yes	No
Sexual Abuse	Category C	Did a person older than you have sexual contact with you?	Yes	No
Household Substance Abuse	Category D	Did you live with anyone who got drunk daily?	Yes	No
		Did you live with anyone who used street drugs?	Yes	No
Household Mental Illness	Category E	Was a household member mentally ill or depressed?	Yes	No
		Did a household member attempt suicide?	Yes	No
Family Member Treated Violently	Category F	Did you witness a family member being pushed, grabbed, or slapped?	Yes	No
		Did you witness a family member being kicked or hit with a fist?	Yes	No
		Were you ever repeatedly hit for at least a few minutes?	Yes	No
		Ever been threatened with or hurt by a gun or knife?	Yes	No
Separation From Parents	Category G	Did you ever live with someone apart from your parents?	Yes	No
Incarcerated Household Member	Category H	Did a household member ever go to prison?	Yes	No
Parental Separation or Divorce	Category I	Were your parents ever separated or divorced?	Yes	No
Bullying	Category J	Did you ever experience bullying in school?	Yes	No
Foster Parents	Category K	Did you live with foster parents?	Yes	No
